# The present and future enhanced recovery after surgery for bladder cancer

**DOI:** 10.1590/S1677-5538.IBJU.2017.0282

**Published:** 2018

**Authors:** Michael A. Poch, Sephalie Patel, Rosemarie Garcia-Getting

**Affiliations:** 1 Department of Genito-Urinary Oncology, Moffitt Cancer Center, Tampa, Florida, United States; 2 Department of Anesthesia, Moffitt Cancer Center, Tampa, Florida, United States

*To the editor,*

Enhanced Recovery After Surgery (ERAS) protocols have been increasingly used in radical cystectomy over the past few years. While the principles were started in colorectal surgery, they can be easily applied to any surgery. The evidence is overwhelmingly in favor of these principles due to improved outcomes such as length of stay, complications, and cost (Table-1) (1-5). Development and implementation of an ERAS protocol requires multidisciplinary involvement, strong participation of everyone involved, and analysis of outcomes. We discuss our experience in development of ERAS for radical cystectomy, barriers to implementation, results at our institution, and future directions.

**Table 1:** Enhanced recovery after surgery - radical cystectomy series.

Author	Type of Study	Comparison	Number of patients	Length of Stay	Complication	Location
	Retrospective	ERAS	71	15	26.7% – 30 day	
Maffezzini (1)						Italy
	Historical Control	pre-ERAS	40	22	22.50%	
Smith (2)						UK
1	Retrospective	ERAS 2	27	7	55.6% – 90 day	
2		ERAS 1	37	10	76.7% – 90 day	
		pre-ERAS	69	14	72.5% – 90 day	
	Historical Control					
	Prospective	ERAS	40	5.2		
Pruthi (3)					not documented	US
	Historical Control	pre-ERAS	30	10		
	Retrospective	ERAS	56	13		
Arumainayagam (4)					30/90 - day not documented	UK
	Historical Control	pre-ERAS	56	17		
	Prospective Observational	ERAS	110	4 Days	65% – 30 day	
Daneshmand (5)						US
	Historical Control - Matched	pre-ERAS				

## Role of Multidisciplinary Approach

We believe the multidisciplinary approach to implementation of an ERAS protocol is crucial for success in patients undergoing radical cystectomy. Prior to starting multidisciplinary ERAS, surgical components of ERAS were already in place with administration of alvimopan, limited use of nasogastric tube, early feeding, and early ambulation. Involvement of our anesthesia colleagues allowed inclusion of anesthetic related ERAS techniques such as multimodal analgesia, limited fasting state, epidural use, and goal directed fluid therapy (GDFT). Our adapted approach to fluid management can be seen in Figure-1. This change translated to involvement with the Pre-Admission Testing clinic, preoperative holding staff, recovery room staff, and floor nursing. Identification of leaders from each discipline is critical to successful implementation of ERAS (6).

Surgical and anesthesia partnership is crucial to ensure the proper functioning of an ERAS protocol. Anesthetic components have been consistently proven to have long-term effects on patient outcomes. This has been recently demonstrated by Jaeger et al. who showed that anesthesiologist experience with radical cystectomy is directly correlated with readmission rate for this procedure (7). Anesthesiologist involvement and accountability is needed in order to include the best evidence based practices within the specialty and to standardize the techniques amongst anesthesia providers.

## Limitations of ERAS

### Study Design

One of the challenges of identifying meaningful differences in clinically important endpoints is lack of using standard criteria. Almost all of the Genitourinary Enhance Recovery studies use length of stay as an endpoint. While this is easy to capture it does not give an adequate representation of clinical recovery. As one could imagine, absolute discharge date is dependent on multiple factors including de mographic logistics, pharmacy readiness and even day of operation (8). The concept of “readiness for discharge” rather than absolute length of stay is likely more relevant and meaningful as a clinical endpoint. Wong-Lun-Hing et al. described 5 specific criteria for discharge or readiness for discharge in patients undergoing ERAS for hepatic surgery (9). These thresholds included regular diet, lack of IV fluid support, oral medication only for pain control, full mobilization, and improving laboratory values; once met, patients were considered “ready for discharge”. In addition to objective criteria for readiness for discharge having independent reviewers to assess whether these criteria are met can remove investigator bias (10).

### Use, efficacy, & perception

While ERAS protocols for radical cystectomy have demonstrated significant improvement in both recovery from surgery and decreasing morbidity, limitations of the protocols and previous studies should be recognized. ERAS studies were initially established for patients undergoing colorectal surgery; modifications such as early feeding were important for patients undergoing large bowel surgery but not necessarily for patients undergoing small bowel surgery, where risk of ileus is substantially lower. It also should be noted that while a majority of the ERAS protocol initiatives implement each element simultaneously, individual element use and adherence is often not reported in the study. We have previously shown that after FDA approval of alvimopan, 13% of patients who were eligible did not receive it (11). This can be seen in the original alvimopan study as well, where only 83% of patients received the medication (12). In addition, in our experience epidural analgesia was not utilized in 100% of patients. Studies evaluating ERAS implementation should be analyzed as both an “intention-to-treat” and “as treated” analysis. Unlike in randomized control trials where-in a single intervention is used, a multi-faceted ERAS program has many moving parts. It's important to distinguish what drives outcomes and how they can be measured. For example, is GDFT more or less important for patients with higher ASA scores or limited ejection fraction? The majority of ERAS studies do not document individual element use (13-15). It should also be noted that while many surgeons and anesthesiologists believe they are using ERAS programs, the reality is far from that. Kukreja et al. reported that only a fraction of the ERAS elements are used among physicians who self-report as ERAS users (16). Among the urologists queried 1, 2 or 3 of the elements were omitted by 13%, 25%, and 23% of the respondents, respectively.

## FUTURE DIRECTIONS

### Preoperative Optimization

After successfully implementing a multidisciplinary ERAS protocol for radical cystectomy, we believe the future lies in providing a more comprehensive approach to perioperative care; as such, we have turned our efforts to expanding the preoperative components of ERAS. The overall goal is to ensure patients are optimized prior to radical cystectomy in order to achieve better postoperative outcomes. This is accomplished by risk stratifying patients and targeting preoperative interventions at modifiable risk factors, such as malnutrition, anemia, and frailty.

The association between poor preoperative nutritional status and increased morbidity and mortality after gastrointestinal surgery is well established (17). Similarly, in urologic surgery malnourished patients undergoing RC have shown higher overall morbidity and 90-day mortality (18). Patients at risk for malnourishment can be identified by various tools, such as the nutritional risk score (NRS). The incidence of malnutrition in patients undergoing RC has been reported up to 19%, making this a promising target for optimization before surgery (18, 19). Providing nutritional support preoperatively to malnourished patients has been shown to reduce incidence of postoperative complications. Immuno-nutrition is a newer nutritional supplement which consists of a mixture of arginine, glutamine, omega 3 fatty acids, and nucleotides taken orally for five days prior to surgery. Though evidence in patients undergoing RC specifically is limited, two pilot studies have shown that preoperative immune-nutrition was associated with fewer postoperative complications, including infections and ileus (20, 21). Munbauhal et al. concluded that immune-nutrition should be considered for malnourished patients undergoing RC beginning 1 week before surgery (22).

Preoperative anemia (PA) is another common surgical risk factor and can be easily diagnosed with routine preoperative laboratory analyses. In patients undergoing radical cystectomy, the prevalence of PA was 40% and associated with worse oncologic outcomes (23). Thus diagnosis and treatment of anemia in the preoperative period is recommended. Optimal treatment in this setting has not been established, however treatment of iron-deficiency anemia with iron infusion may be considered when prompt response is required, as with many oncologic surgeries (24).

Frailty is another reliable predictor of postoperative complications and adverse outcomes, including increased length of stay, discharge to rehabilitation facility, and mortality (25). Though a universal clinical definition is lacking, there are numerous validated frailty assessment tools available. Frailty can be described as an age-related decline in physiologic function and resulting vulnerability to stressors across physical, cognitive, and psychosocial domains, and is estimated to affect 42% of geriatric cancer patients (26-28). Given that two-thirds of urologic surgeries are performed in those >65 years of age, frailty syndrome is likely common in patients undergoing urologic cancer surgery (29). Various interventions have been studied and appear beneficial in reducing frailty and its complications, including physical rehabilitation before surgery (30). Identifying patients at risk for frailty and attempting to optimize functional status before surgery should be considered in patients undergoing RC.

### Minimally Invasive Surgery

Over the past three decades, many studies have demonstrated an improvement in clinical recovery with the incorporation of minimally invasive techniques. These improvements have been demonstrated in post-operative pain scores, length of stay and metabolic stress response to surgery. While the use of minimally and robot-assisted techniques for bladder cancer are still evolving there is certainly potential that perioperative benefits may be seen. There is currently a paucity of data regarding incorporation of ERAS programs for robotic radical cystectomy, however those published demonstrated improvements in length of stay. Unfortunately, those studies evaluating ERAS for robotic cystectomy had varying number of elements use (31). The EAU robotic urology section scientific working group consensus also highlighted the need for “core teams” for operating room staffing. Presence of a “core team” has been shown to improve operating room efficiency and thereby potentially improving outcomes (32). While not unique to robotic surgery, the concept of a “core team” may have higher value in robotics where the primary surgeon is not at bedside. As a general concept, minimally invasive surgery including robotic cystectomy should be seen as a potential additional element to be incorporated into an ERAS program not to be used in lieu of.

### Perioperative Surgical Home

Endorsed by the ASA and American Academy of Orthopedic Surgeons (AAOS), the Perioperative Surgical Home (PSH) is a health care delivery model that has recently gained the attention of the American Urological Association (AUA), which hosted a webinar on the subject in early 2017. The PSH is focused on patient-centered, physician-led, coordinated care from the decision for surgery until the patient has recovered as fully as expected after surgery (33, 34). This model includes anesthesiologist participation in patient care from preoperative optimization to postoperative medical management. While the PSH contains many elements of ERAS, additional emphasis is placed on preoperative interventions to risk stratify and optimize patients, post-discharge follow-up tailored to reduce readmissions, and improved coordination between phases of care. We have learned through design and implementation of ERAS in radical cystectomy at our institution the importance of interdisciplinary collaboration. This has become paramount as we look to expand our care to encompass the full perioperative spectrum, into areas that are not traditionally managed by a single team, or by surgeons and anesthesiologists.

## CONCLUSIONS

Patients undergoing RC benefit from ERAS techniques, as seen in our institution after implementation of a multidisciplinary ERAS protocol with participation from all stakeholders. As we look to further improve clinical outcomes after RC, expansion of preoperative risk assessment and implementation of evidence-based interventions aimed at optimizing high-risk patients are logical next steps. In addition, identifying what ERAS elements provide the highest clinical and financial value, standardized reporting methodologies are paramount. Ultimately, the PSH care model of comprehensive, coordinated care may prove to yield the best clinical results, particularly for complex surgeries such as RC.
